# Covid-19 pandemic and suicidal risk among adolescents

**DOI:** 10.1192/j.eurpsy.2022.1107

**Published:** 2022-09-01

**Authors:** J. Gonçalves Cerejeira, C. Vallecillo Adame, S. Uribe, I. Santos Carrasco, T. Jiménez Aparicio, C. De Andrés Lobo, M. Queipo De Llano De La Viuda, A. Gonzaga Ramírez, G. Guerra Valera

**Affiliations:** 1 Hospital Clínico Universitario de Valladolid, Psychiatry, Valladolid, Spain; 2 Hospital Clínico Universitario, Psiquiatría, Valladolid, Spain; 3 Clinical Hospital of Valladolid, Psychiatry, Valladolid, Spain; 4 Hospital Clínico Universitario de Valladolid, Psiquiatría, VALLADOLID, Spain

**Keywords:** Covid-19, youth, Suicide

## Abstract

**Introduction:**

The Covid-19 pandemic has had a significant impact on the mental health of adolescents. Several descriptive studies and systematic reviews have shown an increase in suicide rates in this age group.

**Objectives:**

- To present a literary review on the impact of the Covid-19 pandemic on the mental health and suicidal behavior of adolescents around the world. - To present data on admission rates due to suicidal behavior during the first year of the Covid-19 pandemic in a Spanish child-adolescent psychiatric hospitalization unit.

**Methods:**

- We will present a literature review and a retrospective cross-sectional study on admission rates for suicidal behavior in a child-adolescent psychiatric hospitalization unit. - Admission rates for suicidal behavior during the year prior to the pandemic will be compared with rates relative to the first year of the pandemic.

**Results:**

- We have found a significant increase in admission rates for suicidal behavior during the year of the pandemic. Similar results have been found in different studies and meta-analyzes. - The socio-demographic characteristics of the patients are quite similar in the two periods of time analyzed, but the reference to intra-family problems has been more frequent in the year of the pandemic.

**Conclusions:**

Our data is in line with other studies suggesting that the Covid-19 pandemic has had a strong impact on teenage suicidal behavior.

**Disclosure:**

No significant relationships.

